# B-Cell RANKL Contributes to Pathogen-Induced Alveolar Bone Loss in an Experimental Periodontitis Mouse Model

**DOI:** 10.3389/fphys.2021.722859

**Published:** 2021-09-14

**Authors:** Rajendra P. Settem, Kiyonobu Honma, Sreedevi Chinthamani, Toshihisa Kawai, Ashu Sharma

**Affiliations:** ^1^Department of Oral Biology, University at Buffalo, Buffalo, NY, United States; ^2^Department of Periodontology, College of Dental Medicine, Nova Southeastern University (NSU), Fort Lauderdale, FL, United States

**Keywords:** periodontitis, alveolar bone loss, RANKL, B-cells, *Tannerella forsythia*

## Abstract

Periodontitis is a bacterially-induced inflammatory disease that leads to tooth loss. It results from the damaging effects of a dysregulated immune response, mediated largely by neutrophils, macrophages, T cells and B cells, on the tooth-supporting tissues including the alveolar bone. Specifically, infiltrating B cells at inflamed gingival sites with an ability to secrete RANKL and inflammatory cytokines are thought to play roles in alveolar bone resorption. However, the direct contribution of B cells in alveolar bone resorption has not been fully appreciated. In this study we sought to define the contribution of RANKL expressing B cells in periodontitis by employing a mouse model of pathogen-induced periodontitis that used conditional knockout mice with B cell-targeted RANKL deletion. Briefly, alveolar bone loss was assessed in the wild-type, B-cell deficient (Jh), or B-cell-RANKL deleted (RANKL^ΔB^) mice orally infected with the periodontal pathogen *Tannerella forsythia*. The RANKL^ΔB^ mice were obtained by crossing Cd19-Cre knock-in mice with mice homozygous for conditional RANKL-flox allele (RANKL^flox/flox^). The alveolar bone resorption was determined by morphometric analysis and osteoclastic activity of the jaw bone. In addition, the bone resorptive potential of the activated effector B cells was assessed *ex vivo*. The data showed that the RANKL producing B cells increased significantly in the *T. forsythia*-infected wild-type mice compared to the sham-infected mice. Moreover, *T. forsythia*-infection induced higher alveolar bone loss in the wild-type and RANKL^flox/flox^ mice compared to infection either in the B cell deficient (Jh) or the B-cell specific RANKL deletion (RANKL^ΔB^) mice. These data established that the oral-pathogen activated B cells contribute significantly to alveolar bone resorption *via* RANKL production.

## Introduction

Periodontitis (PD) is a chronic inflammatory disease affecting over 50% of the United States adult population that results in the loss of tooth supporting tissues including the alveolar bone (Pihlstrom et al., 2005; Darveau, 2010). PD is caused by a dysregulated immune response orchestrated by the supra- and sub-gingival microbes which collectively constitute the oral microbiome (Kumar et al., 2006). PD is also a risk factor for many systemic diseases including diabetes, Alzheimer’s, obesity, and rheumatoid arthritis (Pischon et al., 2007; Seymour et al., 2007). Multiple clinical and experimental studies have shown that both innate and adaptive immune cells (monocytes, macrophages, neutrophils, T and B-cells) play critical roles in the maintenance of periodontal health as well as progression of the disease (Dutzan et al., 2016). Particularly, specific B cell subtypes have been shown to be associated with the disease severity (Nikolajczyk, 2010). Studies have shown that the B cell stimulatory cytokines BLyS (B lymphocyte stimulator) and APRIL (a proliferation-inducing ligand) have been shown to be elevated in periodontitis and required for B cell-dependent alveolar bone loss in a murine model (Abe et al., 2015). Further support for the pathological role of B cells in periodontitis comes from the findings that show that the B cell-deficient mice are protected from *P. gingivalis*-induced alveolar bone loss (Abe et al., 2015; Oliver-Bell et al., 2015). Moreover, studies have shown that the B cells in gingival tissues of individuals with periodontitis express RANKL (the receptor activator of NF-κB ligand), a key osteoclastogenic cytokine (Kawai et al., 2006). A more direct evidence for the role of B cells and their RANKL in periodontitis comes from the studies that showed that bacterially-activated B cells expressed RANKL and adoptive transfer of these cells promoted alveolar bone resorption in a rodent model (Han et al., 2006, 2009). B cells with an anti-inflammatory role also exist in the gingival tissues. For example, a subset of B-regs (B regulatory cells), B10, has been suggested to play anti-inflammatory and anti-bone resorbing functions due its ability to secrete IL-10 (Dai et al., 2017; Hu et al., 2017; Wang et al., 2017), The adoptive transfer of B10 cells in mice has been shown to suppress the RANKL/OPG (osteoprotegerin, a soluble decoy receptor of RANKL) ratio and increase IL-10 production in gingival tissues, and thus these cells may play a regulatory role in osteoclastogenesis (Wang et al., 2017). The bone-damaging effects of B cell secreted RANKL has been shown in other bone diseases as well such as rheumatoid arthritis (Yeo et al., 2011) and osteoporosis (Onal et al., 2012). However, the evidence in favor of B cell-RANKL role in bacterially-induced periodontitis is so far indirect. In our laboratory we study the pathogenesis of the periodontal pathogen *Tannerella forsythia*. Our studies have demonstrated that the TLR2-Th2 inflammatory axis drives the alveolar bone resorption upon *T. forsythia* infection in mice (Myneni et al., 2011).

The purpose of the present study was to determine the contribution of B cell expressed RANKL in periodontitis. We utilized *T. forsythia* as a periodontal pathogen of choice in inducing periodontitis (alveolar bone loss) in a murine model using the wild-type and the conditional knockout mice having B cell specific RANKL deficiency (RANKL^ΔB^). Our results established that RANKL expressing effector B cells are involved in alveolar bone resorption in response to the pathogen infection.

## Experimental Procedures

### Animals and Bacterial Infection

*Tannerella forsythia* was grown in broth or on agar plates (1.5% agar in Broth) under anaerobic conditions as described previously (Chinthamani et al., 2021). Specific-pathogen free wild-type BALB/cJ and C57BL/6J mice, and *CD19-Cre* mice were purchased from Jackson Laboratory (Bar Harbor, ME, United States). B cell deficient mice (Jh; *Igh*^–/–^) were purchased from Taconic Biosciences. Mice harboring a conditional RANKL allele (RANKL-flox) generated previously (Onal et al., 2012) were obtained as gift from Charles O’Brien at the University of Arkansas. B cell-specific RANKL deletion mice were generated by crossing conditional RANKL-floxed mice with CD19-Cre mice by a three-step breeding strategy. Briefly, homozygous *CD19-Cre* (Cre inserted into B cell CD19 gene) transgenic mice were crossed with heterozygous RANKL-flox mice (*RANKL*^*flox/*+^) to generate heterozygous RANKL-flox offspring with and without a Cre allele. These offspring were then intercrossed to generate homozygous RANKL-flox (*RANKL*^*flox/flox*^) mice with and without a Cre allele. Lastly, RANKL^flox/flox^ mice with or without a Cre allele were intercrossed, generating progeny (50%) with the required genotype (homozygous RANKL-flox mice with hemizygous transgenic Cre) and 50% with homozygous RANKL-flox allele serving as littermate controls. Offspring were genotyped by PCR by using the following primer sequences as described before. Cre-for, 5′-GCGGTCTGGCAGTAAAAACTATC-3′; Cre-rev, 5′-GTGAAACAGCATTGCTGTCACTT-3′, product size 102 bp; RANKLflox-for, 5′-CTGGGAGCGCAGGTTAAATA-3′; RANKLflox-rev, 5′-GCCAATAATTAAAATA CTGCAGGAAA-3′, product size 108 bp (wild-type) and 251 bp (floxed allele). The DNA fragment pattern for each genotype is shown in Supplementary Figure 1. Mice were maintained in HEPA-filtered cages with autoclaved food, water, and bedding. Animals (eight male mice per group; housed as 2 littermates per cage) within an experimental group were age matched (*n* = 8, 6–8 weeks old). All procedures were performed in accordance with protocols approved by the University at Buffalo Institutional Animal Care and Use Committee (IACUC). Mice were infected with *T. forsythia* as previously described with following modifications (Myneni et al., 2011). Briefly, mice were first treated with kanamycin (1 mg/mL) in drinking water for 7 days followed by a 3-day antibiotic-free period to suppress resident flora. This was followed by infection with live bacteria (*T. forsythia*) through oral gavage using a feeding needle. Infection was given as 100 μL bacterial suspension (10^10^ c.f.u./mL) in 2% carboxymethyl cellulose (CMC) six times at 48 h intervals. Infection was monitored and confirmed by swabbing gingival tissues around teeth followed by PCR with *T. forsythia* 16S rRNA gene primers (5′-GCGTATGTAACCTGCCCGCA-3′ and 5′-TGCTTCAGTTCAGTTATACCT-3′, amplifying a 641-bp amplicon) as described previously (Settem et al., 2012). The control sham-infected mice received antibiotic pre-treatment and 100 μL of 2% carboxymethyl cellulose only. PCR analysis of oral swabs confirmed that all mice infected with *T. forsythia* were positive for the *T. forsythia*-specific 620-bp 16S rRNA gene product and the sham-infected were negative (data not shown). After 6 weeks of the first infection mice were sacrificed to assess the alveolar bone loss morphometrically, serum antibody response by ELISA and B cell RANKL expression by flow cytometry.

### ELISA for Bacteria-Specific Serum IgG Titers

To determine whether B cell RANKL deficiency had any impact on the antibody response against *T. forsythia*, enzyme-linked immunosorbent assays (ELISAs) were performed as described previously (Settem et al., 2012). Briefly, 96-well Immuno MaxiSorp plates (Nalgene, Rochester, NY, United States) were coated with formalin-fixed *T. forsythia* (1 × 10^8^ cells/well). Sera were added in a two-fold serial dilution, and *T. forsythia*-specific IgG was detected using horseradish peroxidase-conjugated goat anti-mouse IgG (Bethyl Laboratories, Montgomery, TX, United States). ELISA wells were color developed with TMB (3,3′,5,5′-tetramethylbenzidine) microwell enzyme substrate (KPL, Gaithersburg, MD, United States). After stopping the enzyme reaction with 0.1 N H_2_SO_4_, plates were read at 495 nm. The antibody titer was defined as the log_2_ of the highest dilution giving an absorbance 0.1 units above the background.

### Assessment of Alveolar Bone Loss

Mice (*n* = 8) were sacrificed after 6 weeks of the first infection, jaws were autoclaved, de-fleshed and immersed overnight in 3% hydrogen peroxide, and stained with 1% methylene blue. Mouse jaw was then placed horizontally on a platform such that the same plane was analyzed for each mouse. The horizontal bone loss was assessed morphometrically by measuring the distance between the cementoenamel junction (CEJ) and the alveolar bone crest (ABC) at seven buccal sites on the three molars (3, 2 and 2 sites on molars 1, 2, and 3, respectively) on the left side and seven sites on the right side of the maxilla. Measurements at these 14 buccal sites per mouse were made under a stereo zoom microscope (Nikon SMZ1000) with a wide zoom range (0.8×–8×) and a 10x eyepiece, providing an overall magnification of 8×–80×. This microscope was attached to a digital camera (Brook-Anco, Rochester, NY, United States) fitted with an Aquinto imaging measurement system (a4i America). To minimize the effects of any measurement biases, the jaws were independently positioned and read in a random and blinded manner by two evaluators. Total alveolar bone loss per group was calculated by averaging total CEJ-ABC distances (14 sites per mouse) of all mice.

### Flow Cytometric Analysis

#### RANKL Expression on B-Cells

Flow cytometry was performed to assess and compare the level of RANKL expression on B cells from different treatment groups. This analysis was performed to also confirm that conditional deletion of *Rankl* gene in B cells indeed led to the loss of RANKL expression on B cells. For this purpose, spleens and cervical lymph nodes (cLNs) were collected from each mouse at the time of sacrifice and B cells were purified by using the EasySep^TM^ mouse CD19 Positive Selection Kit II (STEMCELL Technologies, MA, United States) as per manufacturer’s instructions. Purified B lymphocytes were cultured in RPMI medium 1640 (Life Technologies, Grand Island) supplemented with 5% heat-inactivated fetal bovine serum (Sigma-Aldrich, St. Louis, MO, United States), 50 μM 2-ME (Sigma-Aldrich), 2 mM L-glutamine,100 U/mL penicillin, and 100 μg/mL streptomycin (Life Technologies) for 48 h. The cells were stained with APC-conjugated rat-anti-mouse CD19 monoclonal antibody (BD Pharmingen, Clone 1D3) and biotin-conjugated rat anti-mouse RANKL monoclonal antibody (eBioscience, Clone IK22/5) followed by streptavidin-FITC (eBioscience). Appropriate rat IgG2a k isotype controls were used as negative controls (eBiosciences). Data were acquired using a Fortessa flow cytometer (BD Biosciences) and the number of positively stained cells in the total counted cells was analyzed for each sample using FCS express (*De Novo* software).

### RT-qPCR Analysis

Reverse transcription quantitative PCR (RT-qPCR) analysis was performed to assess the levels of RANKL mRNA in B cells in response to *T. forsythia* infection. For this purpose, B cells from spleens of sham—and *T. forsythia*—infected mice were purified and then stimulated with *T. forsythia* at an m.o.i. of 50 and 100 for 48 h. After stimulation, toral RNA was isolated from B cells with an RNAeasy mini kit (Qiagen) incorporating DNase treatment as per the manufacturer’s protocol. Retrotranscription of RNA (500 ng) into cDNA was performed with iScript reverse transcriptase kit (Bio-Rad laboratories). Quantitative real-time PCR was performed with a Bio-Rad iCycler (Bio-Rad) using SYBR Green master mix reagent (Bio-Rad). Two step PCR was performed with 94°C for 15 s and 58°C for 30 s for 40 cycles. Gene expression values were calculated based on the 2^–ΔΔCt^ method using *Gapdh* expression as an internal control. The primer sequences were: GAPDH, 5′-GGATGCAGGGATGATGTTCT-3′ and 5′-AACTTTGCCATTGTGGAAGG-3′; RANKL, 5′-AGCCATTTGCACACCTCAC-3′ and 5′-CGTGGTACCAAGA GGACAGAGT-3′.

### TRAP Staining

### *Ex vivo* Osteoclastogenesis

The ability of activated B cells to induce osteoclastogenesis *via* RANKL was evaluated by an *ex vivo* assay utilizing TRAP (tartrate-resistant acid phosphatase) based staining of osteoclasts. Wild type BALB/cJ mice were treated with Kanamycin (1 mg/mL) in drinking water for 1 week to suppress the natural flora effect. After 3 days of antibiotic-free water mice were infected with 100 μL of *T. forsythia* at 1 × 10^10^ c.f.u./mL per dose for a total of six doses given at 48 h interval. Sham group received 100 μL of 2% CMC alone. After 2 weeks of the first infection mice were sacrificed and CD19^+^ B cells were isolated with CD19 positive selection kit (Stemcell Technologies) as per the manufacturer instructions. The CD19^+^ cells were primed with *T. forsythia* for 3 days at an m.o.i. of 10 and 50. The primed B cells were washed twice with PBS and co-cultured with mouse bone marrow derived macrophages (BMMs) for 3 days. In some conditions B cell and macrophage co-cultures were incubated in the presence of mouse OPG-Fc at a concentration of 10 μg/mL to confirm that the osteoclastogenesis is mediated by RANKL-RANK interaction. TRAP (tartrate-resistant acid phosphatase) staining for detecting osteoclasts was performed as described previously (Settem et al., 2013). TRAP-positive cells with three or more nuclei were considered as matured osteoclasts and counted microscopically.

#### *In vivo* Osteoclastgenesis

For evaluation of *in vivo* osteoclastic activity, mouse jaw bone sections were TRAP stained. Mice (C57BL6, RANKL^ΔB^ and RANKL^flox/flox^) maxillary and mandibular jaw bones (*n* = 4) were fixed in 10% phosphate-buffered formalin and decalcified in 10% EDTA. The samples were embedded in paraffin, and sections at 4 μm were prepared and TRAP stained. The stained slides were digitally scanned with a Scan Scope CS system (Aperio) immediately to minimize color fading. The scanned slides were viewed with Image Scope viewing software (Aperio). The right maxillary and mandibular inter-dental areas (average of 10 higher power fields/slide) of the crestal alveolar bone from the first molar to third molar were used to quantify osteoclasts.

### Data Analysis

Prism 9 software (GraphPad Software) was used for all statistical analyses. Statistical significance was determined by two-tailed paired or unpaired Student’s *t* test for two groups or one-way ANOVA with Tukey’s *post hoc* test for multiple groups. Comparisons of two non-parametric data sets were done by the Mann–Whitney *U* test. A *p* value of less than 0.05 was considered statistically significant.

## Results

### B Cell Deficiency Attenuates Pathogen Induced Alveolar Bone Loss

**FIGURE 1:**
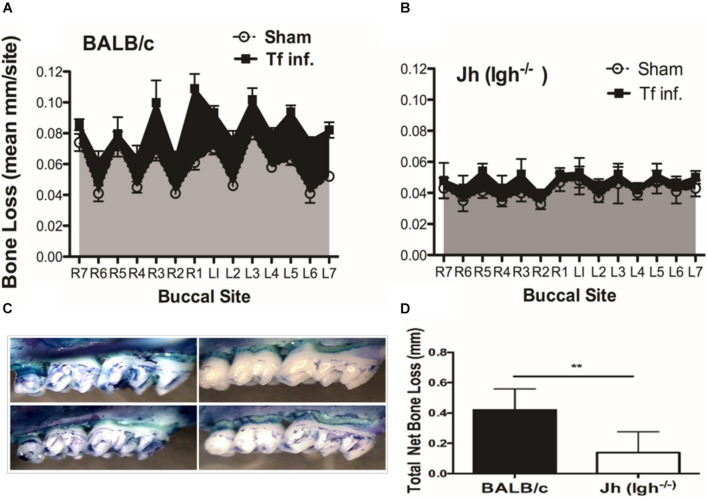
B cell deficiency attenuates pathogen induced alveolar bone loss. Wild type and Jh (Igh^–/–^) mice (*n* = 8) were infected by oral gavage either with *T. forsythia* cells (Tf. Inf) mixed in carboxymethyl cellulose (CMC) or CMC alone (Sham). Alveolar bone destruction was assessed after 6 weeks by measuring the distance from the ABC to the CEJ at 14 maxillary buccal sites per mouse (R1 to R7, right jaw; L1 to L7, left jaw). Average alveolar bone loss at 14 buccal sites wild-type **(A)** and Jh (mature B-cell deficient) mice **(B)**. **(C)** Representative images of maxillary jaw bones showing alveolar bone levels at each site measured as distances between ABC to CEJ. **(D)** Net bone loss calculated as average of total bone loss in *T. forsythia* infected group—total bone loss in sham group) in wild-type and Jh (Igh*^–/–^*) group. As indicated, the net bone loss in the B cell deficient mice is significantly lower as compared to the wild-type mice. Data show means with standard deviations. Statistically significant differences are indicated with asterisks. p–values less than 0.05 were considered significant. (***P < 0.001; *P < 0.05).

To evaluate the impact of B cell deficiency on pathogen-induced alveolar bone loss, 6 weeks after the first *T. forsythia* infection alveolar bone resorption was measured at 14 buccal sites of left and right (7 + 7) maxillary jaw bones. Because cementum is progressively exposed with increasing bone resorption, the distance between the CEJ to ABC was determined as a measure of bone resorption at each site. As shown in Figure 1A the wild-type mice showed a significant elevation in bone resorption at all sites measured when infected with *T. forsythia* (Figure 1C, top right panel) as compared to sham infection (Figure 1C, top left panel). On the other hand, B cell deficient Jh (*Igh*^–/–^) mice exhibited reduced alveolar bone resorption at all molar sites in response to *T. forsythia* infection (Figure 1C, bottom left, sham; bottom right, Tf infection) as compared to the sham infection (Figure 1B). As seen in Figure 1D the total net bone loss due to *T. forsythia* infection was significantly higher in the wild-type BALB/cJ as compared to the Jh (*Igh*^–/–^) mice. These data showed that *T. forsythia*-induced bone loss, similar to that by the oral pathogen *P. gingivalis* shown previously, requires mediation of B cells (Abe et al., 2015; Oliver-Bell et al., 2015). These data confirmed that mature B-cells play a significant role in the pathogen induced alveolar bone resorption.

### *Tannerella forsythia* Induces RANKL Expression on B Cells

**FIGURE 2:**
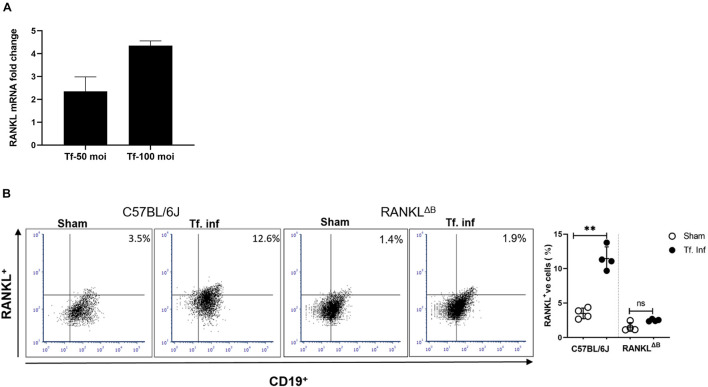
*T. forsythia* infection induces RANKL expression on B cells. Expression levels of RANKL in mouse B cells. *T. forsythia* infected mouse spleen B cells were purified and re-stimulated with *T. forsythia* at an MOI of 50 and 100 for 48 h. After re-stimulation, RANKL mRNA and B cell surface expression levels were determined by RT-qPCR **(A)** and flow cytometry **(B)**, respectively. **(A)** Data show fold-increase in RANKL expression in B cells from *T. forsythia*-infected mice versus sham-infected mice. **(B)** Representative flow cytometry plots of draining cLN B cells showing dual RANKL/CD19 positive cells in the upper right quadrant. Bar graphs on the right show mean (±s.d) percentages of RANKL producing cells in one of two experiments with four mice in each group. Data are presented as means ± s.d. (*n* = 4 animals/group). ^∗∗^*P* < 0.01.

RANKL expression in B cells was assessed by analyzing B cells from spleens and cervical draining lymph nodes (cLN) after infection. B cells were purified from the wild-type or conditional knockout (cKO) mice with B cell specific RANKL deletion (RANKL^ΔB^) 6 weeks after the first *T. forsythia*—or sham- infection dose. Purified splenic B cells (CD19^+^) were activated *in vitro* for 48 h with *T. forsythia* at an m.o.i. (multiplicity of infection) of 10 and 50. The RT-qPCR data showed a significant fold-increase in RANKL mRNA expression in B cells from *T. forsythia*-infected wild-type (C57BL/6J) mice in comparison to sham mice (control) (Figure 2A). There was no measurable RANKL mRNA expression in B cells from RANKL^ΔB^ mice, sham- or *T. forsythia*—infected (Data not shown; C_t_ values were over the range set at 40 cycles). In addition, the flow cytometry data showed that *T. forsythia*-infected wild-type mice had higher percentages of RANKL^+^ B cells as compared to the sham-infected mice. On the other hand, RANKL^ΔB^ mice exhibited no significant increase in RANKL^+^ B cell population following infection and the percentages of RANKL^+^ B cells remained low. As shown, *T. forsythia*-infected wild-type mice alone showed increased CD19^+^/RANKL^+^ cell population (Figure 2B).

### B Cell RANKL Contributes to *T. forsythia*-Induced Osteoclastogenesis

**FIGURE 3:**
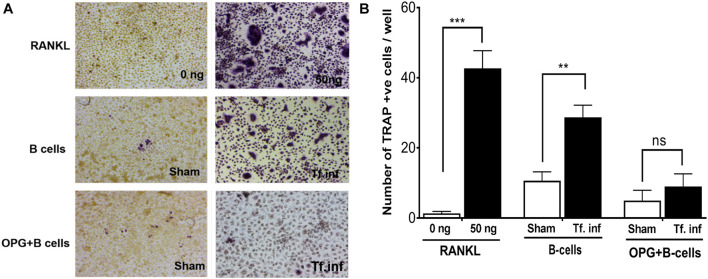
*T. forsythia* causes osteoclastogenesis *via* induction of RANKL^+^ effector B cells. Evaluation of osteoclastogenesis *in vitro* by TRAP staining. CD19^+^ve B cells from mice were co-cultured with bone marrow derived macrophages. In some condition’s co-cultures were performed in the presence of mouse OPG-Fc at a concentration of 10 μg/mL to confirm the osteoclastogenesis is mediated by RANKL-RANK interaction. TRAP staining was performed to determine the number of multinucleated osteoclasts. **(A)** Representative histological staining showing increased multinucleated TRAP positive cells (magenta-red) in cocultures of bone marrow derived macrophages with B cells from *T. forsythia* infected (Tf. Inf) versus sham infected mice. **(B)** Data are means ± s.d. of TRAP^+^ cells per well for each treatment from a representative experiment performed with three replicates per condition. Inclusion of OPG in cocultures significantly reduced the number of TRAP positive cells. **p < 0.01; ***p < 0.001.

We assessed the contribution of RANKL induction in B cells due to activation by *T. forsythia* on osteoclastogenesis. For this purpose, purified splenic B cells from the sham- or *T. forsythia-* infected wild-type mice were first activated with *T. forsythia* and then co-incubated with mouse bone marrow derived macrophages. The formation of multinucleated osteoclasts was evaluated by TRAP (tartrate-resistant acid phosphatase) staining. The data showed significantly increased numbers of TRAP positive cells in culture containing B cells from *T. forsythia*-infected animals as compared to those from the sham-infected mice (Figures 3A,B). In addition, the number of multinucleated TRAP positive cells diminished significantly in the presence of osteoprotegerin (OPG, a RANKL decoy receptor), thereby validating that the ability of activated B cells to promote osteoclast formation was mediated *via* RANKL.

### Deletion of RANKL on B Cell Attenuates Pathogen Induced Alveolar Bone Loss

**FIGURE 4:**
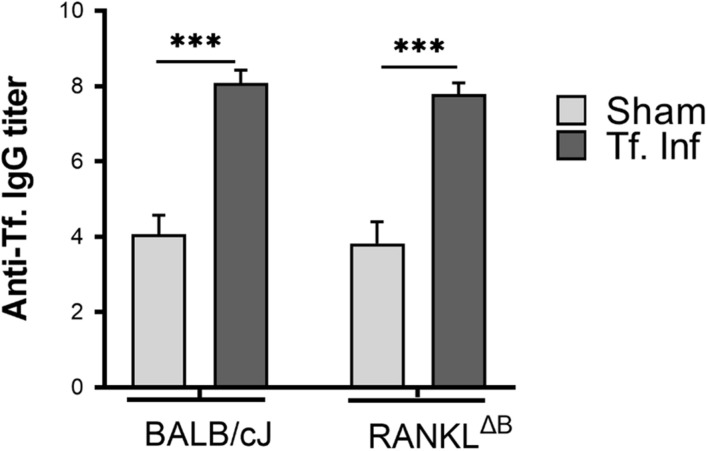
Deletion of RANKL in B cells did not alter the IgG response. Sera was collected from sham and *T. forsythia* infected mice (BALB/cJ and RANKL^ΔB^) and analyzed for *T. forsythia*-specific IgG by ELISA. Antibody levels are presented as log_2_ titers. Data are the means and standard deviations for each group (*n* = 8); ^∗∗∗^*P* < 0.001 vs the sham-infected group.

**FIGURE 5:**
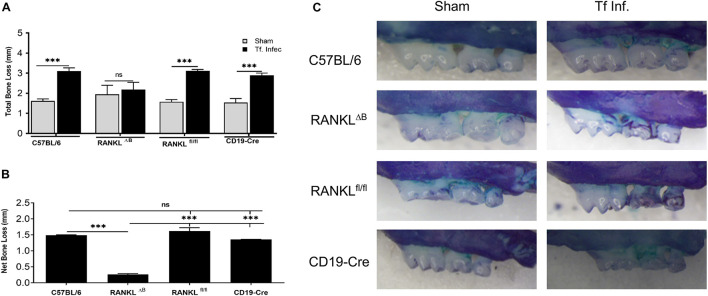
Deletion of RANKL in B cells attenuates *T. forsythia* induced alveolar bone loss. Mice were infected by oral gavage with 6 doses (10^9^ cells/dose) of either *T. forsythia* (10^9^ cells/dose; Tf WT) or sham-infected and alveolar bone levels were determined. **(A)** Alveolar bone destruction was assessed by measuring the distance from the ABC to the CEJ at 14 maxillary buccal sites per mouse (*n* = 8). **(A)** Total alveolar bone loss per group was calculated by averaging total CEJ-ABC distances (14 sites per mouse) of all mice. **(B)** The net bone loss, calculated as mean total ABC-CEJ distance of *T. forsythia* infected group minus mean total ABC-CEJ distance of the sham-infected group, show that the net bone loss caused by *T. forsythia* is significantly higher in RANKL^fl/fl^ mice than RANKL^ΔB^ mice. **(C)** Representative images of maxillae from each group stained with methylene blue and imaged with a Nikon SMZ 1000 microscope (original magnification X3). Bars indicate means and standard deviations. ^∗∗∗^*p* < 0.001.

**FIGURE 6:**
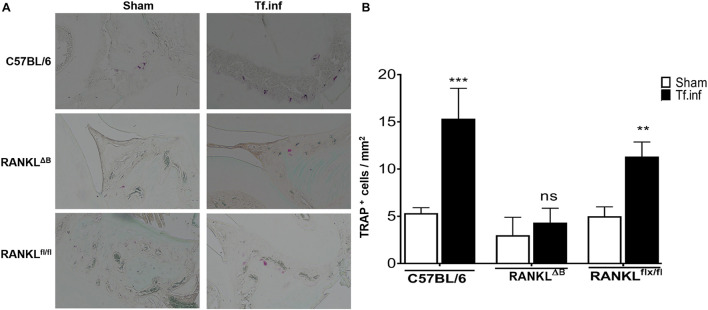
Alveolar bone loss correlates with osteoclastic activity. **(A)** Representative histological sections showing TRAP^+^ cells from *T. forsythia* (Tf. Inf) and sham (Sham) infected mice (×400). **(B)** Average number of TRAP^+^ cells in 10 high-power magnification fields/slide (*n* = 4 mice/group). **p < 0.01, ***p < 0.001; Tf. Inf vs Sham.

Next, alveolar bone resorption in response to oral infection by *T forsythia* in the wild-type and B cell specific RANKL deletion mice (RANKL^ΔB^) was compared to confirm the role of B cell produced RANKL in the pathogen-induced alveolar bone resorption. Before analyzing the alveolar bone loss in mice, we assessed the antibody response to *T. forsythia* in both the wild-type and conditional KO mice to confirm that the B cells’ ability to mount an antibody response was not compromised in any way due to RANKL deletion. The data showed (Figure 4) a significant elevation of serum antibody titers against *T. forsythia* in both the wild-type and RANKL^ΔB^ mice infected with *T. forsythia* as compared to the respective sham-infected mice. These data suggested that RANKL did not impact the antibody response of B cells. The antibody titers in sham-infected animals against *T. forsythia* represent cross-reactive anti-bacterial antibodies induced by the mouse resident flora. Next, the total and net alveolar bone loss associated with the maxillary jaw bones was calculated as described above. As shown in Figure 5A, total alveolar bone loss in the wild-type mice infected with *T. forsythia* was significantly higher as compared to that in the sham-infected wild-type mice. On the other hand, the *T. forsythia* infected RANKL^ΔB^ mice exhibited only a marginal but non-significant increase in bone loss. This marginal increase in bone loos could be attributed to RANKL expression in other cell types as such as the T cells and neutrophils as has been previously shown. Both the homozygous flox and the CD19 heterozygous genotype mice infected with *T. forsythia* showed a significant alveolar bone loss similar to the *T. forsythia* infected wild-type mice. The net bone loss for each group (total average alveolar bone loss of the sham-infected mice subtracted from that of the *T. forsythia*-infected mice). As is evident from the data (Figure 5B), *T. forsythia* induced similar levels of net alveolar bone loss in the wild-type, RANKL^flox/flox^, or CD19-Cre heterozygous mice but the RANKL^ΔB^ mice were significantly resistant (Figure 5C, panels showing representative maxillae from each group). The alveolar bone loss observed was confirmed by evaluation of the osteoclastic activity in the jaw bones by TRAP staining. The TRAP staining results showed increased numbers of TRAP positive cells in *T. forsythia* infected wild-type, RANKL^flox/flox^ and CD19-Cre mice as compared to the RANKL^ΔB^ mice (Figure 6). Taken together, these data demonstrated that RANKL production by B cells contributes significantly to pathogen infection induced osteoclastogenesis in the jaw bone, culminating in alveolar bone loss in mice.

## Discussion

In this study we demonstrated that the mature B cell deficient mice and mice deficient specifically in B cell associated RANKL (cKO RANKL^ΔB^) were resistant to pathogen-induced alveolar bone loss. These data confirm that activated B cells *via* RANKL expression contribute significantly to pathogen associated alveolar bone loss observed in periodontitis. Previous studies have shown that activated T and B cells in gingival tissues of individuals with periodontitis express RANKL (receptor activator of nuclear factor κB ligand, also known as TNFSF11) (Taubman et al., 2005; Han et al., 2006, 2009; Kawai et al., 2006). RANKL is a tumor necrosis factor (TNF) family inflammatory cytokine which is a key positive inducer of osteoclastogenesis due its role in the differentiation, survival, and activation of osteoclasts (Boyce and Xing, 2007; Nakashima et al., 2012). In light of the fact that mice with B cell specific RANKL deficiency exhibited some pathogen-induced alveolar bone loss, albeit minimally, indicates that other RANKL expressing cells in addition to the B cells are likely also involved in driving alveolar bone loss. Nevertheless, our data establishes a significant role and contribution of RANKL expressing effector B cells in driving alveolar bone loss during periodontitis.

In periodontitis, various subtypes of T (Th1, Th2, Th17, and Tregs) and B (plasma cells, B1, B2, and B- regs) lymphocytes with inflammatory or anti-inflammatory properties play a crucial roles in modulating periodontal inflammation and bone loss (Figueredo et al., 2019). In heath and disease, the proportion of each of these subtypes in the gingival tissues has been shown to differs. For instance, with regard to B cells, lower numbers of memory B cells are present in periodontitis lesions compared to gingivitis and the numbers of antibody-secreting CD138^+^ plasma cells increase in periodontitis compared to healthy or gingivitis tissues (Mahanonda et al., 2016). A recent study showed that RANKL expression increases in all subsets of activated B cell in severe periodontitis (Demoersman et al., 2018). However, the mechanisms by which RANKL expression in B lymphocytes is induced are unclear. Nevertheless, IL-33 has been implicated in the activation of RANKL expression in T and B cells during periodontitis (Malcolm et al., 2015). It was demonstrated that IL-33 treatment along with pathogen infection can significantly increase the population of RANKL-expressing T and B cells compared to pathogen infection alone. Strikingly, IL-33 is an inducer of Th2 cells (Murakami-Satsutani et al., 2014) and a regulator of B cell proliferation and differentiation (Komai-Koma et al., 2011; Stier et al., 2019). How these effects of IL-33 might impact periodontitis remains to be determined. Moreover, B cells in the gingival tissues can also produce inflammatory cytokines (IL-1β and TNF-α) in response to TLR ligands (Jagannathan et al., 2009), that in turn can induce RANKL expression and inflammation to cause alveolar bone loss. We have previously shown that TLR2-Th2 axis is involved in driving the *T. forsythia* induced alveolar bone loss in mice. However, how this axis is linked to B cell RANKL induction remains to be determined. It is tempting to speculate that in addition to the RANKL induction *via* TLR2 pathway in B cells mentioned above, Th2 cytokine IL-4 might help to promote B cell proliferation and maturation (Granato et al., 2014).

B cells with an anti-inflammatory role have also been reported in gingival tissues. A subset of B-regs (B regulatory cells), B10, has been found in gingival tissues of patients with and without periodontitis, and has been suggested to play anti-inflammatory and anti-bone resorbing functions due its ability to secrete IL-10 (Dai et al., 2017; Hu et al., 2017; Wang et al., 2017). B10 cells by virtue of IL-10 secretion can potentially regulate osteoclastogenesis by inhibiting the secretion of TNF-α and IL-1β and suppressing the ratio of RANKL/OPG in gingival tissues (Wang et al., 2017). The cKO mouse model for B cell RANKL described in our study serves as a model system for evaluation of the contribution of other B cell cytokines in periodontitis. For instance, the role of B cell secreted OPG (Tnfrsf11b) or 1L-10 can be interrogated by conditional deletion of respective alleles in B cells by employing *Tnfrsf11b*^flox/flox^ or *IL-10*^flox/flox^ mice, respectively, bred with CD19-Cre mice.

In summary, we have shown by utilizing B cell-specific conditional knockout mice that activated B cells are significant contributors of RANKL in the oral pathogen-induced alveolar bone loss. However, what kind of B cell subtype(s) becomes RANKL^+^ effector cell in the gingival tissue in response to the pathogen could not be ascertained with the current study, Finally, our findings indicate that the B cell cKO mouse model described here for RANKL can be adapted for defining the contribution of other B cell mediators in periodontitis progression.

## Data Availability Statement

The original contributions presented in the study are included in the article/Supplementary Material, further inquiries can be directed to the corresponding author/s.

## Ethics Statement

The animal study was reviewed and approved by IACUC, University at Buffalo.

## Author Contributions

AS conceived the work. AS and RS wrote the manuscript. RS, KH, and SC performed the experiments. AS, RS, and TK critically analyzed the data and all authors were critical reviewers of the manuscript.

## Author Disclaimer

The content is solely the responsibility of the authors and does not represent the official views of the NIDCR or the NIH.

## Conflict of Interest

The authors declare that the research was conducted in the absence of any commercial or financial relationships that could be construed as a potential conflict of interest.

## Publisher’s Note

All claims expressed in this article are solely those of the authors and do not necessarily represent those of their affiliated organizations, or those of the publisher, the editors and the reviewers. Any product that may be evaluated in this article, or claim that may be made by its manufacturer, is not guaranteed or endorsed by the publisher.

## Supplementary Material

Supplementary Figure 1 Electrophoretic patterns of PCR products from different mouse genotypes.
