# Supplementary figures and images for: B-Cell RANKL Contributes to Pathogen-Induced Alveolar Bone Loss in an Experimental Periodontitis Mouse Model
Source: Front Physiol. 2021 Sep 14;12:722859. doi: 10.3389/fphys.2021.722859 (PMC8476884; doi:10.3389/fphys.2021.722859)

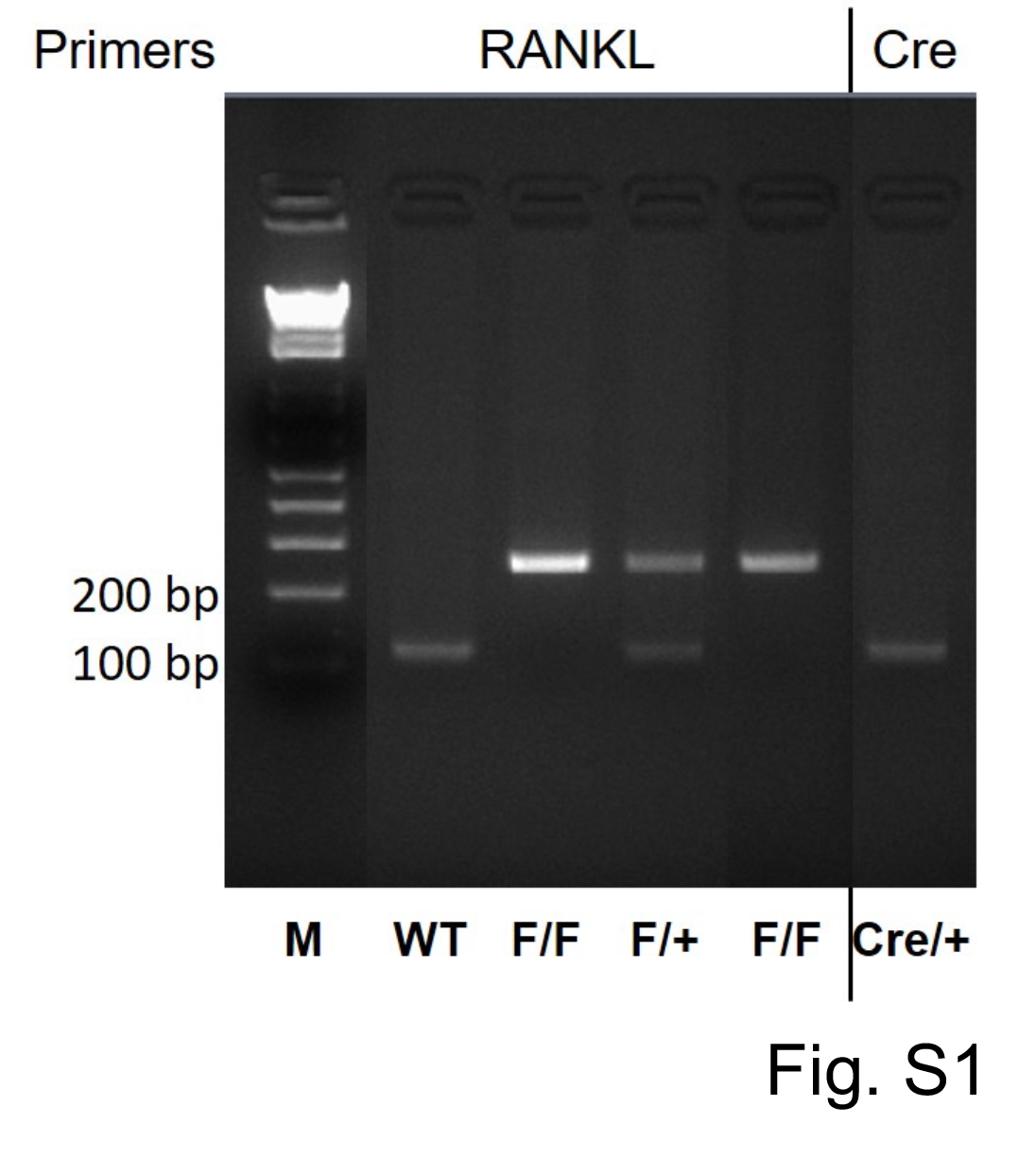

Supplement: Supplementary Figure 1 — Electrophoretic patterns of PCR products from different mouse genotypes. [file Image_1.TIF]
